# Significantly impaired shoulder function in the first years of rheumatoid arthritis: a controlled study

**DOI:** 10.1186/s13075-015-0777-0

**Published:** 2015-09-20

**Authors:** Annelie Bilberg, Tomas Bremell, Istvan Balogh, Kaisa Mannerkorpi

**Affiliations:** Institute of Medicine, Department of Rheumatology and Inflammation Research, Sahlgrenska Academy, University of Gothenburg, Guldhedsgatan 10, Box 480, 40530 Göteborg, Sweden; Institute of laboratory Medicine, Department of Occupational and Environmental Medicine, University of Lund, 22185 Lund, Sweden; Institute of Neuroscience and Physiology, Section of Health and Rehabilitation, Physiotherapy, Sahlgrenska Academy, University of Gothenburg, Box 455, 40530 Göteborg, Sweden

## Abstract

**Introduction:**

Patients with rheumatoid arthritis (RA) risk impaired shoulder function due to the inflammatory process. The knowledge of shoulder function in the early years of the disease is limited. The aim was to compare shoulder function and activity limitation related to the shoulder-arm-hand in women with RA in early disease course compared to age-matched healthy women.

**Method:**

This controlled cross-sectional study included 103 women with rheumatoid arthritis and a reference group of 103 age-matched healthy women. The mean age was 47.1 (SD 10.0) years, the mean disease duration was 20.3 (SD 8.5) months and the mean DAS28 score was 3.8 (SD 1.4) among the patients. Participants were provided with self-reported questionnaires quantifying activity limitations. Shoulder function was assessed by isometric strength of the shoulder, shoulder-arm movement and shoulder pain. Hand-grip force was assessed and examination was made of tender and swollen joints among the patients.

**Results:**

Patients showed significantly (p < 0.0001) impaired shoulder muscle strength, shoulder-arm movement, and shoulder pain compared to the reference group. Patients shoulder muscle strength was approximately 65 % of the results observed in the reference group. Activity limitations related to the shoulder-arm-hand (DASH) were significantly (p < 0.0001) higher in the patient group compared to the reference group and indicates limitations in daily activities for the patients.

**Conclusion:**

Patients with RA were found to have significantly impaired shoulder function already 1.5 years after disease onset compared to age-matched subjects. Reduced shoulder muscle strength was found to be associated with activity limitations (DASH) implying that screening of the shoulder function, emphasising the shoulder muscle strength, should be initiated from disease onset.

**Electronic supplementary material:**

The online version of this article (doi:10.1186/s13075-015-0777-0) contains supplementary material, which is available to authorized users.

## Introduction

The shoulder is the third most common site of musculoskeletal pain in the general population [1] and the prevalence of shoulder symptoms has been reported to be somewhere between 7 and 27 % [2, 3]. Patients with rheumatoid arthritis (RA) have an additional risk of impaired shoulder function as a consequence of inflammation. Synovitis, bursitis and tendinitis causes decreased muscle strength, persistent pain, reduced range of motion and joint destruction, which may lead to functional loss and difficulties with daily activities. Shoulder function correlates with activity limitations in patients with RA [4–7] where pain, decreased muscle strength, a reduced active range of motion and the disease activity itself have been suggested to contribute to the limitation.

Traditionally, shoulder joint involvement in RA is considered to apply to patients with long-term disease [8–10] and to patients who are older at onset [11, 12]. Reduced range of shoulder motion and shoulder muscle strength is common among patients with established RA [5]. However, little is known about shoulder function in the early disease course. To our knowledge, shoulder function has only been sparsely studied with a focus on shoulder movement [13]. Because impaired shoulder function also occurs in the general adult population, we found it interesting to compare patients with RA in an early phase of the disease with a gender-matched and age-matched reference group of self-reported healthy individuals.

The aim of this study was to compare different aspects of shoulder function in women with RA during the first years of disease with that in an age-matched reference group of self-reported healthy women to show the impact of RA on the shoulder. Our hypothesis was that shoulder function in RA is reduced early in the disease course.

## Methods

### Design

A multicentre, controlled cross-sectional study was conducted in the Region of Västra Götaland, West Sweden.

### Patient group selection

Eligible patients were women aged 20–60 years who met the 1987 American College of Rheumatology criteria for RA, with a disease duration ranging from 6 months to 3 years. Exclusion criteria were other severe and chronic somatic or psychiatric diseases, shoulder arthroplasty, any unhealed fracture of the upper extremities, ongoing adhesive capsulitis or inability to read and speak Swedish. Patients were recruited from three rheumatology units at Sahlgrenska University Hospital, Skövde Hospital and Uddevalla Hospital following a search of the Swedish RA register and a review of the medical records of patients with RA from 2006 to 2008. One hundred and forty-three women were identified and invited to participate in the study; however, 13 patients did not meet the inclusion criteria due to other rheumatic diseases, not understanding the Swedish language or other severe concomitant disease. A further 27 patients could not be enrolled due to time restrictions or a lack of contact, or declined to participate, leaving a total of 103 patients. The study population has previously been included in another study of ours [14]. Because men have greater variability than women with regard to shoulder muscle strength, a reference group of men would require quite a large number of participants. Therefore no men were included in the study.

Information about demographic data and disease variables was obtained in interviews and from the patients’ medical records. Examinations and administration of the questionnaires were carried out by four experienced physical therapists. Examinations of joints for assessment of tender and swollen joints in the patients were conducted under the supervision of an experienced rheumatologist. More than 70 % of the patients were rheumatoid factor (RF) positive and anti-cyclic citrullinated peptide (anti-CCP) positive and almost 40 % showed erosive changes within 2 years of disease duration. This indicates that the study population is representative of other RA populations in Scandinavia with a disease duration ranging from 6 months to 3 years [15, 16].

### Reference group selection

A reference group was recruited through a newspaper advertisement and from the public sector in Gothenburg, selected according to age. Eligible participants were self-reported healthy women, aged 20–60 years. Exclusion criteria were the same as for the patient group, with an additional exclusion criterion of RA. Subjective shoulder symptoms such as pain did not lead to exclusion from participation in the reference group since 10.5–23.8 % of the general female population in Sweden report pain from the shoulder–upper arm [17]. Participants with shoulder arthroplasty, unhealed fracture of the upper extremities and ongoing adhesive capsulitis were excluded because of the inability to perform the physical performance-based tests correctly without risking the person’s condition.

One hundred and twenty-one women who met the selection criteria and volunteered to participate were, after the assessments, individually matched for age with the patients by a computer program for randomisation, leaving a total of 103 participants in each group.

Information about demographic data, possible diseases and shoulder trauma was obtained by interview. Examinations and administration of the questionnaires were carried out by two experienced physical therapists.

Written and oral study information was provided to all study participants and written consent was obtained. The study was approved by the Regional Ethical Review Board in Gothenburg, Sweden.

### Measurements

Shoulder function was assessed using three variables: muscle strength of the shoulder, active shoulder–arm movement and shoulder pain during movement.

The isometric muscle strength of the shoulder abductor muscles was assessed with an ISOBEX 3.0 dynamometer (Cursor AG, Bern, Switzerland) [18], which measures isometric strength in kilograms. Strength was recorded in a seated position and the tested arm in lateral elevation of 90° in the scapular plane. A strap from the dynamometer, attached to the floor, was placed proximal to the wrist. The patient was instructed to elevate the arm from the original position as much as possible for 5 seconds. The best performance out of three was selected [19].

Active shoulder–arm movement was assessed with the shoulder–arm movement impairment instrument [4, 20], measuring hand raising, hand to opposite shoulder, hand behind back, hand to neck and hand to seat. The score ranges from 1 to 6 (full ability) and the total sum score ranges from 5 to 30.

Shoulder pain during shoulder and arm movements was assessed by the Borg symptom scale [21]. The score ranges from 0 (no pain) to 10, and the total sum score ranges from 0 to 50.

Hand-grip force was assessed by a digital electronic dynamometer, the Grippit (AB Detektor Gothenburg, Sweden) [22, 23], which measures the grip force in Newtons. The device displays the best, the mean and the end value for the hand grip force for each test round. The mean grip force was used for assessment.

Activity limitations related to the shoulder–arm–hand were assessed with the Disabilities of the Arm, Shoulder and Hand (DASH) questionnaire [24, 25], a self-administered questionnaire to assess upper extremity disability and symptoms comprising 30 items [1–5] concerning the patient’s health status during the preceding week. The total score ranges from 0 to 100 (severe disability):

$$ \mathrm{DASH}\ \mathrm{score} = \left(\left(\mathrm{sum}\ \mathrm{o}\mathrm{f}\ \mathrm{all}\ \mathrm{points}\ \mathrm{f}\mathrm{o}\mathrm{r}\ \mathrm{all}\ \mathrm{the}\ \mathrm{questions}\ \hbox{-}\ 30\right)/1.2\right). $$

The mean DASH score for norm values for a general US population aged 19–75+ is 10 (standard deviation (SD) 15) [26].

General activity limitations among the patients were assessed with the Health Assessment Questionnaire (HAQ) Index [27, 28], which is a RA disease-specific instrument that measures eight aspects of activity during the previous week rated from 0 to 3 (severe difficulties). The total mean score is calculated from the eight aspects.

Disease activity was recorded by the Disease Activity Score of 28 joints (DAS28) [29] and is based on a calculation of the erythrocyte sedimentation rate (mm/hour), the number of swollen and tender joints (28-joint index) and self-reported general health scored on a visual analogue scale (0–100). A higher value indicates more disease activity.

RF and anti-CCP for the patients were assessed with standard laboratory tests at the accredited laboratories of Sahlgrenska University Hospital.

The occurrence of erosions was assessed by radiographs of the hands, wrists and feet. The presence of erosions is a marker of disease severity.

Physical workload was assessed by type of work categories using a classification system [30]. The categories were ‘heavy material handling’, ‘heavy repetitive’, ‘medium-heavy load’, ‘light repetitive’ and ‘administration/computer work’.

Physical activity during leisure time was assessed with the Leisure Time Physical Activity Instrument (LTPAI), which assesses the amount of physical activity during a typical week [31].

### Statistical methods

Descriptive data are presented as mean (SD) or median (range) and data for categorical variables as number (percentage). For comparison between RA patients and the reference group, the Mann–Whitney *U* test was used for continuous variables, the Mantel–Haenzel chi-square test for ordered categorical variables and Fisher’s exact test for dichotomous variables. Calculations were made of 95 % confidence intervals for differences between the means.

The Wilcoxon signed-rank test was used for comparison of dominant and non-dominant arms. The Spearman correlation coefficient was used for the correlation analysis. The Mann–Whitney *U* test was used for comparisons of continuous variables between patients and healthy subjects reporting shoulder symptoms and those reporting no symptoms. All significance tests were two-sided and conducted at the 5 % significance level. Power analysis demonstrated that, with the sample size of 103 in each group, we would achieve a power of 0.97 to detect a 20 % difference in shoulder strength between the patient and reference groups based on the Mann–Whitney *U* test with α = 0.05.

## Results

Characteristics of the patient group are presented in Table 1. The mean age of the patients was 47.1 (SD 10.0) years and of the participants in the reference group 47.1 (SD 10.1) years. The mean duration of disease was 20.3 (SD 8.5) months and the mean DAS28 was 3.8 (SD 1.4) for the patients (see Table 2). The large majority of the patients were RF positive (78.6 %) and anti-CCP positive (75.5 %). Radiographs were performed at an average of 21.1 (SD 10.1) months after diagnosis, showing erosive changes of the hands and/or feet in 38 % of the patients. Radiographs were not performed in four patients owing to administrative difficulties. In the patient group, 73 % worked compared with 97 % in the reference group, and the mean working hours per week was significantly (*p* <0.0001) lower in the patient group (24.3 (SD 16.8) hours) than in the reference group (36.8 (SD 6.9) hours). Twenty-eight (27 %) of the patients did not work at all due to sick-leave, disability pension or unemployment. There were no significant differences with regard to physical workload between the patient group and the reference group. Patients were significantly (*p* = 0.003) less physically active during leisure time compared with the reference group (see Table 1).

**Table 1:** Characteristics of the study population

Variable	Patient group (*n* = 103)	Reference group (*n* = 103)	*p* value
Age (years)	47.1 (10.0)	47.0 (10.1)	0.97
	49.0 (20.0; 60.0)	49.0 (21.0; 60.0)	
Education			<0.0001
<10 years	27 (26.5 %)	4 (3.9 %)	
10–12 years	41 (40.2 %)	20 (19.6 %)	
>12 years	34 (33.3 %)	78 (76.5 %)	
Dominant hand, right	93 (90.3 %)	101 (98.1 %)	0.033
Work hours per week	24.3 (16.8)	36.8 (6.9)	<0.0001
	30.0 (0.0; 40.0)	40.0 (0.0; 40.0)	
	*n* = 101	*n* = 96	
Work status			<0.0001
Full-time 80–100 %	49 (47.6 %)	89 (86.4 %)	
Part-time, 1–79 %	26 (25.2 %)	11 (10.7 %)	
Non-working, 0 %	28 (27.2 %)	3 (2.9 %)	
Workload			0.61
Heavy load	3 (2.9 %)	1 (1.0 %)	
Medium-heavy load	39 (38.2 %)	40 (40.0 %)	
Light repetitive	9 (8.8 %)	4 (4.0 %)	
Administration/computer	51 (50.0 %)	55 (55.0 %)	
Leisure-time activity (hours)	6.34 (4.11)	8.52 (5.47)	0.0032
	6.00 (0.00; 20.00)	7.00 (1.00; 26.00)	
	*n* = 97	*n* = 103	

For categorical variables, number (percentage) is presented. For continuous variables, mean (standard deviation) or median (minimum; maximum) per participant is presented

**Table 2:** Disease characteristics of the patients

Variable	Patients (*n* = 103)
Disease duration (months)	20.3 (8.5)
	19.0 (6.0; 36.0)
DAS28, 0–10	3.82 (1.37)
	3.87 (1.00; 7.41)
	*n* = 96
Rheumatoid factor positive, yes	81 (78.6 %)
Anti-CCP, yes	74 (75.5 %)
	*n* = 98
Rheumatoid arthritis erosions, yes	39 (39.8 %)
DMARDs	91 (89.2 %)
Anti-TNF treatment	14 (13.6 %)
Health Assessment Questionnaire, 0–3	0.60 (0.55)
	0.50 (0.00; 2.63)
	*n* = 102

For categorical variables, number (percentage) is presented. For continuous variables, mean (standard deviation) or median (minimum; maximum) per participant is presented

*anti-CCP* anti-cyclic citrullinated peptide, *DAS28* DAS28, Disease Activity Score of 28 joints, *DMARD* disease-modifying anti-rheumatic drug, *TNF* tumour necrosis factor

### Shoulder symptoms

At the time of the assessment, 53.4 % of the patients and 20.4 % in the reference group reported present shoulder symptoms. Thirty-three (32.0 %) patients had unilateral symptoms and 22 (21.4 %) bilateral symptoms. In the reference group, unilateral shoulder symptoms were found to be more common compared with bilateral symptoms.

### Shoulder function

The majority of the study population reported right-hand dominance, 90.3 % of the patients and 98.1 % of the healthy subjects. There was a significant (*p* = 0.033) difference between the groups regarding right-hand dominance, and analyses of the shoulder function and hand-grip force were therefore conducted according to the dominant arm and the non-dominant arm. No significant differences between the dominant and non-dominant arms for shoulder strength were found in the patient group (mean difference 0.11 (SD 0.82), *p* = 0.091) or in the reference group (mean difference 0.03 (SD 0.84), *p* = 0.56). Hence, only the dominant arm is presented in the Results.

Patients showed significantly (*p* <0.0001) impaired shoulder function with regard to shoulder strength, shoulder–arm movement, lateral shoulder elevation and shoulder pain compared with the reference group for the dominant arm (see Table 3).

**Table 3:** Assessments of shoulder muscle strength, shoulder movement shoulder pain, hand-grip force and the DASH questionnaire in the patient and reference groups

Variable	Patient group (*n* = 103)	Reference group (*n* = 103)	Difference between groups^a^	*p* value
Shoulder strength (kg), 5 seconds d. arm	3.67 (1.64)	5.59 (1.22)	–1.92 (–2.32; –1.52)	<0.0001
	3.60 (0.40; 7.70)	5.40 (3.10; 9.90)		
	*n* = 102	*n* = 103		
Shoulder abduction (°), d. arm	164.3 (23.1)	178.7 (4.1)	–14.4 (–19.0; –9.7)	<0.0001
	170.0 (45.0; 180.0)	180.0 (160.0; 180.0)		
	*n* = 101	*n* = 101		
Shoulder–arm movement, 5–30, d. arm	27.4 (2.9)	29.7 (0.7)	–2.29 (–2.87; –1.72)	<0.0001
	28.0 (11.0; 30.0)	30.0 (26.0; 30.0)		
Shoulder pain, 0–50, d. arm	7.63 (7.12)	0.88 (2.14)	6.80 (5.35; 8.24)	<0.0001
	6.00 (0.00; 38.00)	0.00 (0.00; 12.00)		
Hand-grip force (N), 10 seconds d. arm	159 (78.2)	288 (60.0)	–128 (–147; –109)	<0.0001
	151 (16.0; 350)	294 (157; 441)		
DASH, 0–100	25.7 (17.3)	2.63 (5.36)	23.1 (19.5; 26.6)	<0.0001
	21.3 (0.0; 73.3)	0.83 (0.0; 26.7)		
	*n* = 102	*n* = 102		

For continuous variables, mean (standard deviation) or median (minimum; maximum) per participant is presented For comparison between groups, the Mann–Whitney *U* test was used for continuous variables

^a^Mean (95 % confidence interval)

*d* dominant, *DASH* Disability of the Shoulder, Arm and Hand

### Shoulder muscle strength

The mean isometric shoulder strength for the dominant arm in the patient group (3.7 kg (SD 1.6)) was significantly (*p* <0.0001) lower compared with the reference group (5.6 kg (SD 1.2)) (see Table 3).

### Active shoulder–arm movement

The mean lateral shoulder elevation for the dominant arm in the patient group (164.3° (SD 23.1)) was significantly (p <0.0001) lower than that in the reference group (178.7° (SD 4.1)). The mean active shoulder–arm movement for the dominant arm in the patient group (27.4 (SD 2.9)) was significantly (*p* <0.0001) lower than in the reference group (29.7 (SD 0.7)) (see Table 3).

### Shoulder pain during movement

The mean shoulder pain was significantly (*p* <0.0001) higher for the patients’ dominant arm (7.6 (SD 7.1)) compared with the reference group (0.9 (SD 2.14)) (see Table 3).

### Hand-grip force

The mean hand-grip force in the dominant arm in the patient group (159 N (SD 78)) was significantly (*p* <0.0001) lower than in the reference group (288 N (SD 60)) (see Table 3).

Significant differences were found between the dominant and non-dominant hands for hand-grip force in the patient group (mean differences 10.9 N (SD 47.3), *p* = 0.008) and in the reference group (mean differences 22.0 N (SD 33.3), *p* <0.0001).

### Activity limitations of the shoulder–arm–hand

Activity limitations related to the shoulder–arm–hand (DASH questionnaire) were significantly (*p* <0.0001) higher in the patient group (25.7 (SD 17.3)) than in the reference group (2.6 (SD 5.4)) (see Table 3).

**Fig. 1:**
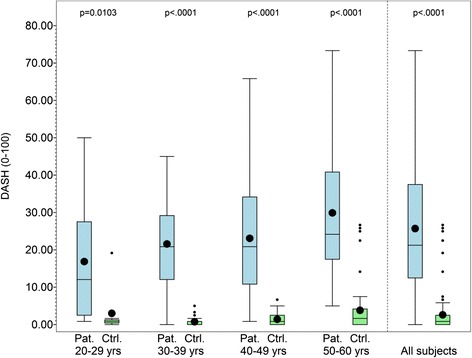
Box plot of DASH score by age group for the patient and reference groups, and for the total study population. A significant difference between the groups was found for all age groups for the DASH score. *Ctrl.* control, *DASH* Disability of the Arm, Shoulder and Hand, *Pat.* patient

The DASH score was significantly higher for the patients in all age groups compared with the reference group when the groups were divided into 10-year age intervals (see Fig. 1).

### Associations between shoulder muscle strength and physical assessments and disease activity in the patient group

The associations between the shoulder muscle strength of the dominant arm and shoulder pain (*r*_s_ = –0.48, <0.001), hand-grip force (*r*_s_ = 0.51, *p* <0.001), DAS28 (*r*_s_ = –0.34, *p* = 0.001) and DASH score (*r*_s_ = –0.45, *p* <0.001) were all found to be significant.

### Group comparison between patients and healthy subjects reporting shoulder symptoms and those reporting no shoulder symptoms

Patients reporting shoulder symptoms showed significantly (*p* <0.0001) impaired shoulder function with regard to shoulder strength, shoulder–arm movement, lateral shoulder elevation and shoulder pain for the symptomatic shoulder when compared with healthy subjects reporting shoulder symptoms. Significant impaired shoulder function for both the dominant and non-dominant arms was also found in patients reporting no shoulder symptoms when compared with healthy subjects reporting no shoulder symptoms (Additional file 1).

## Discussion

The aim of this controlled, cross-sectional study was to compare the shoulder function and activity limitations related to the shoulder–arm–hand in women with RA in the first years of disease with an age-matched reference group of self-reported healthy women.

In patients, the shoulder function was found to be significantly impaired for all of the shoulder variables studied—shoulder muscle strength, active shoulder–arm movement and shoulder pain during movement—when compared with the reference group. Moreover, patients reported significantly more activity limitations (DASH questionnaire) compared with the reference group, indicating limitations of daily activities.

Shoulder muscle strength in the patient group was approximately 65 % of the strength in the reference group. The impaired shoulder muscle strength in the patient group corresponds to previous findings of reduced general muscle strength in patients with longstanding RA [23, 32, 33]. However, our results indicate that shoulder muscle strength is reduced already at an early stage of disease. A possible contributing factor to the reduced shoulder muscle strength might have been the increased shoulder pain during movement in the patient group that was found to correlate significantly with the shoulder muscle strength. It has previously been suggested that functional ability is more influenced by disease activity than by joint destruction in early RA [34, 35]. The significant correlation found between shoulder muscle strength and DAS28 in the present study supports previous studies reporting associations between muscle strength and inflammatory disease parameters in RA [36, 37]. The anatomic origin of the reduced shoulder muscle strength is not targeted in our study. However, periarticular and soft tissue engagements of the shoulder have been reported previously in painful RA shoulders [8]. Moreover, early fatty degeneration of the rotator cuff [9], induced by periods of pain and inactivity, might be an additional contributing factor to the reduced shoulder muscle strength in our patient group as well as asymptomatic rotator cuff tears [38]. Furthermore, the lower overall leisure-time physical activity level reported in the patient group might also have contributed to the reduced shoulder muscle strength.

An unexpected result was the reduction of shoulder muscle strength to 73 % in patients reporting no shoulder symptoms compared with healthy subjects reporting no shoulder symptoms. The impaired shoulder function in patients reporting no shoulder symptoms indicates that patients are not always aware of their functional limitations. This finding shows the importance of initiating screening of the shoulder function at the time of disease onset in all patients, not just among those reporting shoulder problems.

Although shoulder movement was found to be significantly reduced in the patient group as compared with the reference group, the majority of the patients appear to have sufficient shoulder movement for daily activities.

Compared with the reference group, patients’ hand-grip force was reduced to approximately 55 %, corresponding to a previous study in early RA [39]. Hand-grip force has been suggested to correlate with muscle strength in the upper extremities [40], which our result supports since we found a moderate association (*r*_s_ = 0.50) between the two outcome measures. However, we find it important to assess both hand-grip force and shoulder muscle strength when screening for possible impairments of the upper extremities among patients with RA in the first years of disease.

The majority of the patients reported some degree of activity limitations as assessed with the DASH questionnaire compared with the reference group. In addition, when compared with the norm values suggested by Hunsaker et al. [26], our patients showed a higher DASH score in all age groups. On the other hand, the mean HAQ score was found to be low in the patient group, indicating low activity limitations [41]. These results might be seen as inconsistent. However, the DASH questionnaire appear to contain questions concerning more physical strenuous activities than does the HAQ and may better reflect the demands of daily living in physically active patients with RA. We have previously validated the DASH questionnaire for Swedish patients with RA and have found that the DASH score correlated well with the DAS28 and the HAQ score as well as with shoulder function variables and hand-grip force [6]. The DASH questionnaire seems to be appropriate for screening activity limitations of the upper extremity in RA and can be a complement to the HAQ.

For an accurate and objective evaluation of the shoulder function in patients it is important to compare with an age-matched and gender-matched healthy reference group. The patient group and the reference group did not differ in age or physical workloads, which are both found to be strong predictors of shoulder symptoms in the general population [3, 42, 43]. However, significant differences were found for education, work status and leisure-time physical activities, where the reference group had a higher education level, worked a greater number of hours per week and had a higher leisure-time physical activity level. These findings were expected since low education level [44], work disability [45, 46] and low leisure-time physical activity level [47] are common in RA patients. However, the reference group appears to have a slightly higher education level compared with the general population [48]. The prevalence of shoulder symptoms in the general population has been found to be somewhere between 7 and 27 % [2, 3], which is in agreement with a previous Swedish study of shoulder–upper arm pain in the general female population [17]. The variation has been suggested to be explained partly by the differences in the definition of shoulder symptoms and the methods used for its estimation [3]. Our findings are in agreement with these previous studies because 20 % of the healthy women in the reference group reported shoulder symptoms [2, 3, 17].

Furthermore, our reference values for shoulder muscle strength are consistent with those of a previous study of norm values for isometric shoulder muscle strength in healthy subjects [38].

The well-matched reference group in terms of age, gender and physical workload is a strength of the study. However, this was a controlled cross-sectional study and the causality of impaired shoulder function, disease activity and activity limitations cannot be stated. In future studies, a prospective follow-up of patients with newly onset RA with regard to shoulder function are warranted. Such a study would provide an opportunity to identify the patients at risk of shoulder dysfunction and help to explain the natural progression of the disease and its impact on shoulder function. Moreover, to improve our anatomical understanding of the impaired shoulder function in the early disease course, radiographic and ultrasound images seem important to be assessed.

## Conclusions

The overall results of this study indicate that patients with RA have reduced shoulder muscle strength and limited function already 1.5 years after disease onset, even if they do not complain of symptoms from the shoulder. Shoulder muscle strength is related to activity limitations (DASH questionnaire), grip strength, shoulder pain and measures of disease activity (DAS28). Patients would benefit from assessment of shoulder function early in the disease course because of implications for therapy.

## Additional file

Additional file 1:
**Is a table presenting assessments of shoulder muscle strength, shoulder movement shoulder pain, hand-grip force and the DASH questionnaire in the patients and reference group reporting and not reporting shoulder symptoms.** For continuous variables mean (SD) or median (minimum; maximum) / *n* is presented. For comparison between groups the Mann–Whitney *U* test was used for continuous variables. Shoulder function and hand-grip force for patients and references reporting shoulder symptoms are presented for the symptomatic arm (*n* = 15) or if bilateral symptoms for the dominant arm (*n* = 27). Shoulder function and hand-grip force for patients and references reporting no shoulder symptoms are presented for the dominant arm. (DOC 35 kb)

## Abbreviations

anti-CCP: Anti-cyclic citrullinated peptide
DAS28: Disease activity score of 28 joints
DASH: Disabilities of the arm, shoulder and hand
HAQ: Health assessment questionnaire
LTPAI: Leisure time physical activity instrument
RA: Rheumatoid arthritis
RF: Rheumatoid factor
SD: Standard deviation
