# Supplementary material for: Significantly impaired shoulder function in the first years of rheumatoid arthritis: a controlled study
Source: Arthritis Res Ther. 2015 Sep 20;17(1):261. doi: 10.1186/s13075-015-0777-0 (PMC4575780; doi:10.1186/s13075-015-0777-0)
Supplement: Additional file 1: — Is a table presenting assessments of shoulder muscle strength, shoulder movement shoulder pain, hand-grip force and the DASH questionnaire in the patients and reference group reporting and not reporting shoulder symptoms. For continuous variables mean (SD) or median (minimum; maximum) / n is presented. For comparison between groups the Mann–Whitney U test was used for continuous variables. Shoulder function and hand-grip force for patients and references reporting shoulder symptoms are presented for the symptomatic arm (n = 15) or if bilateral symptoms for the dominant arm (n = 27). Shoulder function and hand-grip force for patients and references reporting no shoulder symptoms are presented for the dominant arm. (DOC 35 kb) [file 13075_2015_777_MOESM1_ESM.doc]

**Assessments of shoulder muscle strength, shoulder movement shoulder pain, hand-grip force and the Disability of the Shoulder, Arm and Hand (DASH) questionnaire in the patients and reference group reporting and not reporting shoulder symptoms**

| **Variable** | **Patients reporting**  **shoulder symptoms (n=54)** | **References reporting**  **shoulder symptoms (n=21)** | **p-value** | **Patients not reporting shoulder symptoms**  **(n=49)** | **References not reporting shoulder symptoms**  **(n=82)** | **p-value** |
| --- | --- | --- | --- | --- | --- | --- |
| **Age, years** | 48.9(9.8) | 50.3(8.6) | 0.607 | 44.9(9.9) | 46.0(10.4) | 0.441 |
| **Shoulder strength (kg), 5s ,** | 3.1(2.9)  n=53 | 4.9(1.0) | <0.0001 | 4.2(1.5) | 5.7(1.2) | <0.0001 |
| **Shoulder abduction (degrees),** | 167.7.(20.9) (n=52) | 178.0(5.2)  (n=20) | 0.002 | 167.2(25.7)  (n=48) | 178.2(4.5)  (n=81) | <0.0001 |
| **Shoulder-arm movement, 5-30,** | 26.4(3.3) | 29.3(1.3) | <0.0001 | 28.4(1.7) | 29.7(0.6)  n=82 | <0.0001 |
| **Shoulder pain, 0-50,** | 10.20(8.27) | 2.37(3.41) | <0.0001 | 5.03(4.26) | 0.48(1.41) | <0.0001 |
| **Hand-grip force (N), 10s,** | 154.5(89.8) | 275.6(59.9) | <0.0001 | 162.0(58.8) | 288.8(59.7) | <0.0001 |
| **DASH, 0-100** | 31.5(19.4)  n=53 | 6.6(7.9) | <0.0001 | 19.2(11.9) | 1.6(3.9)  n=78 | <0.0001 |
| For continuous variables Mean (SD) / Median (Min; Max) / n= is presented.For comparison between groups the Mann Whitney U-test was used for continuous variables. | | | | | | |
